# Residential Altitude Associates With Endurance but Not Muscle Power in Young Swiss Men

**DOI:** 10.3389/fphys.2020.00860

**Published:** 2020-07-23

**Authors:** Norina N. Gassmann, Katarina L. Matthes, Patrick Eppenberger, Marek Brabec, Radoslav Panczak, Marcel Zwahlen, Nicole Bender, Thomas Wyss, Frank J. Rühli, Kaspar Staub

**Affiliations:** ^1^Institute of Evolutionary Medicine, University of Zurich, Zurich, Switzerland; ^2^Zurich Center for Integrative Human Physiology (ZIHP), University of Zurich, Zurich, Switzerland; ^3^Institute of Veterinary Physiology, Vetsuisse Faculty, University of Zurich, Zurich, Switzerland; ^4^Institute of Computer Science of the Czech Academy of Sciences, Prague, Czechia; ^5^School of Earth and Environmental Sciences, The University of Queensland, Brisbane, QL, Australia; ^6^Institute of Social and Preventive Medicine, University of Bern, Bern, Switzerland; ^7^Swiss Federal Institute of Sport Magglingen SFISM, Magglingen, Switzerland

**Keywords:** general additive models, VO_2max_, hemoglobin, C-reactive protein, multiple imputation, Switzerland

## Abstract

**Introduction:**

Physical fitness benefits health. However, there is a research gap on how physical fitness, particularly aerobic endurance capacity and muscle power, is influenced by residential altitude, blood parameters, weight, and other cofactors in a population living at low to moderate altitudes (300–2100 masl).

**Materials and Methods:**

We explored how endurance and muscle power performance changes with residential altitude, Body Mass Index (BMI), hemoglobin and creatinine levels among 108,677 Swiss men aged 18–22 years (covering >90% of Swiss birth cohorts) conscripted to the Swiss Armed Forces between 2007 and 2012. The test battery included a blood test of about 65%, a physical evaluation of about 85%, and the BMI of all conscripts.

**Results:**

Residential altitude was significantly associated with endurance (*p* < 0.001) but not with muscle power performance (*p* = 0.858) after adjusting for all available cofactors. Higher BMI showed the greatest negative association with both endurance and muscle power performance. For muscle power performance, the association with creatinine levels was significant. Elevated C-reactive protein (CRP) and hemoglobin levels were stronger contributors in explaining endurance than muscle power performance.

**Conclusion:**

We found a significant association between low to moderate residential altitude and aerobic endurance capacity even after adjustment for hemoglobin, creatinine, BMI and sociodemographic factors. Non-assessed factors such as vitamin D levels, air pollution, and lifestyle aspects may explain the presented remaining association partially and could also be associated with residential altitude. Monitoring the health and fitness of young people and their determinants is important and of practical concern for disease prevention and public health implications.

## Introduction

Physical fitness is beneficial for health and longevity (Kyu et al., 2016). Physical fitness is influenced by and associated with excess body weight, physical activity behavior, lifestyle, motivation, socio-demographic factors (such as socioeconomic background and urbanicity), sex, age, genetics and more factors (Wyss et al., 2007; Schmidt et al., 2017). A set of either health- or skill-related attributes define physical fitness (Caspersen et al., 1985), which is considered to be a multifaceted composite of cardiorespiratory endurance, muscle strength, muscle power, agility (speed), flexibility, and balance (Wyss et al., 2007; Leyk et al., 2015). Aerobic endurance is (clinically) relevant because of its negative correlation with cardiovascular risk factors (Kind et al., 2019), while better muscle power is linked to balance, for example preventing disability in the elderly (Cadore et al., 2014; Muehlbauer et al., 2015). Physical fitness positively correlates with physical activity, which is defined as any production of bodily movement by skeletal muscles with the result of energy expenditure (Caspersen et al., 1985). From a public health perspective, physical inactivity is still a major problem (Federal Office of Public Health, 2019). Consequently, monitoring the health and fitness status of young people is valuable because the earlier that risk factors can be identified and modified, the more favorable morbidity and mortality outcomes become later in life (Engeland et al., 2003). To measure physical fitness, physical performance tests can be used (Wyss et al., 2007).

On the one hand, selected blood parameters levels are associated with physical exercise (Fragala et al., 2017). And on the other hand, excess weight [mostly indicated by Body Mass Index (BMI)] associates with selected blood parameters and reflects an individual’s health status and physical fitness (Staub et al., 2018). Frequently measured blood parameters related to physical exercise and performance include hemoglobin (Hb), creatinine and C-reactive protein (CRP) (Kanstrup and Ekblom, 1984; Kao et al., 2006; Mairbäurl et al., 2013; Fragala et al., 2017). For example, an increase of total Hb leads to a significant improvement in aerobic endurance performance, whereas a decrease of Hb results in significant reduction of performance (Mairbäurl et al., 2013). Creatinine serum levels on the other hand are expected to be higher in individuals with higher muscle mass and active individuals due to a higher creatine turnover in the skeletal muscle, as creatinine is produced after the breakdown of creatine-phosphate and released into the circulation (Fragala et al., 2017). Disease-related inflammation is also associated with lower physical activity (and vice versa), and can be quantified by CRP (Kao et al., 2006; Fragala et al., 2017).

Another factor influencing metabolic parameters and physical performance is residential altitude, with even moderate altitudes influencing hemoglobin levels (Sharma et al., 2019; Staub et al., 2020). The oxygen sensing system of renal cells is triggered by hypoxemia that in turn induces erythropoietin expression. Once it reaches the circulation, erythropoietin is transported to the bone marrow where it stimulates red blood cell proliferation and maturation (Gassmann and Muckenthaler, 2015). Elevated red blood cell mass increases the oxygen transport capacity and ultimately leads to improvement in aerobic endurance (Stray-Gundersen et al., 2001). In addition, there is evidence that muscle power is also positively influenced by altitude, which could be explained among others by reduced aerodynamic resistance in reduced air density or by modification of motor unit recruitment patterns (Feriche et al., 2017).

There is a gap in our knowledge regarding as to how physical performance (in particular aerobic endurance and muscle power) is influenced by low to moderate residential altitude, metabolic parameters, excess weight and other cofactors. Switzerland is an ideal testing ground for such studies because the nation includes geographical areas at low to moderate residential altitudes (where data for broadly varying residential altitudes are much more readily available) and provides high quality conscription data (Panczak et al., 2014). These data cover more than 90% of annual birth cohorts of Swiss men and were previously analyzed separately in the context of excess weight (Panczak et al., 2014), metabolic parameters (Staub et al., 2018), physical performance (Wyss et al., 2019), and altitude (Staub et al., 2020). Here we combined, for the first time, high-quality data from the aerobic endurance and muscle power tests with anthropometric data and blood parameters from the medical examination, which also includes socio-demographic data such as the altitude of the place of residence, occupational background and level of urbanization (urbanicity) of over 100,000 young Swiss men.

As presented in our recent work (Staub et al., 2020), we found a persistent impact of residential altitude on elevated Hb levels among the Swiss conscripts. Based on these data, we now go one step further, and hypothesize that young men living at moderate altitude perform better both in aerobic endurance and in muscle power tests compared to young men living at lower altitude. We also assess the importance of other explanatory co-factors like metabolic parameters, excess weight, or socio-demographic aspects, and we analyze how much of the variation in endurance and muscle power performance can be explained by all these variables.

## Materials and Methods

### Swiss Conscription

The conscription process for the Swiss Armed Forces is described in details elsewhere (Panczak et al., 2014; Bruggisser et al., 2016). All young men with Swiss citizenship are called up for conscription at approximately 19 years of age by the Swiss Armed Forces. This call includes those young men whose military services are later deferred or who are exempted. Hence, generally more than 90% of a given Swiss male birth cohort are represented annually by these conscription data (Panczak et al., 2014). The detailed medical assessment is a compulsory part of the conscription process. Between 5 and 10% of the conscripts per year are assessed as unfit for military service without physically attending the conscription process. Those men suffer from severe diseases and or have severe physical and psychiatric disabilities (Panczak et al., 2014).

### Medical Examination and Socio-Demographic Data

The present study includes *N* = 108,677 conscripts who attended their first, regular assessment between January 2007 and December 2012. Under contractual agreement with the study authors, the Swiss Armed Forces (Armed Forces Staff) provided the fully anonymized data (Staub et al., 2018). The delivered original data included age group (1-year intervals), height (cm), weight (kg), current occupation (as a free-text entry), name and postal (ZIP) code of place of residence, recruitment center and date, Hb (g/l), CRP (in mg/l), and creatinine (μmol/l). Height and weight measurements were taken without shoes but in underwear by medical personnel. We excluded conscripts who were older than 22 years of age when they attended their first, regular assessment with long delay (Staub et al., 2018). Lausanne, Sumiswald, Monte Ceneri, Windisch, Rüti, and Mels are the six centers where conscription takes place. The technical equipment and general organization are identical in all centers and conducted by medical professionals (Panczak et al., 2014). Since the Monte Ceneri, Windisch and Rüti recruitment centers do not cover areas with elevated residential altitude, we only included the Lausanne, Sumiswald and Mels recruitment centers in this study (see Supplementary Figures S1, S2, flowchart and maps).

#### Metabolic Data

The conscripts are asked to participate in a voluntary laboratory blood test, to which approximately 65% consent (Bruggisser et al., 2016). During all the years, blood samples were sent to a single laboratory (Viollier AG) where they are tested, usually within 12 h, using state-of-the art equipment and assays. During the whole observation period, the laboratory underwent regular evaluation and validation processes by internal and external quality controls to ensure identical measurement standards. The measuring devices were produced by Siemens Healthcare Diagnostics AG, Zurich, Switzerland.

The blood tests included Hb, creatinine and CRP, as well as other parameters (Staub et al., 2018). Hb was measured on a Siemens Advia 120, creatinine and CRP on an Advia 1650 (Bruggisser et al., 2016). We chose to analyze these three blood parameters for the following reasons: An increase or decrease of hemoglobin has a significant impact on physical performance (Mairbäurl et al., 2013) and altitude influences hemoglobin (Staub et al., 2020). Higher serum creatinine is found in athletic populations. This is also expected for individuals with a high muscle mass (Fragala et al., 2017). The association between higher physical activity and lower CRP has previously been reported (Fragala et al., 2017). CRP is produced by the liver and is an inflammatory factor that increases due to inflammation or infection (Fragala et al., 2017). CRP is commonly used as a blood parameter to evaluate ongoing inflammation and overall assessment of health (Kao et al., 2006). We excluded 28 values in total, 8 values of Hb < 101 g/l and 20 values of creatinine >149 μmol/l (Manrai et al., 2018). We created a binary indicator of inflammation based on CRP level [CRP >= 5.0 mg/l = 1 (inflammation) and CRP < 5.0 mg/l = 0 (no inflammation)] in order to control for possible impact of inflammation (Hahn and Alscher, 2018).

#### Socio-Economic Background

The conscripts’ socio-economic categories were determined based on their professions, which were assigned to the Socio-Economic Index of Occupational Status (ISEI- 08). ISEI-08 makes it possible to compare occupations according to their socio-economic status and is based on information on the income, education and occupations of almost 200,000 men and women from 42 countries (Ganzeboom and Treiman, 2019). The ISEI distribution of the occupations was divided into three equal groups: lower tertile (ISEI values 12–28), middle tertile (ISEI values 29–43), and upper tertile (ISEI values 44–89). Together with the pupils/students and the group without occupation or with insufficient information, a total of 5 occupational groups [socioeconomic position (SEP) Groups] were included in the analysis.

#### Residential Altitude

The postal (ZIP) codes of places of residence were standardized to the state of March 31 2013, as there are changes of a postal (ZIP) code area over time. We used high-resolution topographical model of the Federal Office of Topography^1^ to derive altitude [in meters above sea level (masl)]. Because we did not have the full residential addresses due to data protection, the residential altitude was approximated using the mean altitude of all 1-m resolution geographic coordinates of residential buildings. We included all buildings within the postal code that at the time of December 31, 2012 had resident males aged (18–25) listed in the registry-based census [data from the Swiss National Cohort SNC^2^, (Bopp et al., 2009)] per postal (ZIP) code. Only for descriptive analysis, residential altitude was grouped in categories of 300 masl according to previous studies on health and altitude in Switzerland (Faeh et al., 2016).

### Fitness Test Data

In addition to the above parameters, we used the data from the fitness performance test developed for the recruitment of the Swiss Armed Forces (Wyss et al., 2007). Approximately 85% of the conscripts who present in the conscription centers are physically evaluated (Bruggisser et al., 2016; Staub et al., 2018). The Swiss Armed Forces fitness test battery consists of five tests described in detail in Wyss et al. (2007). We chose two of them: the progressive endurance run (PER), which allows aerobic endurance capacity to be measured, and the standing long jump (SLJ) to show muscle power of the lower extremities (Wyss et al., 2019). Regarding cardiorespiratory endurance, measuring the maximal oxygen uptake (VO_2max_) correlates more with cardiovascular risk factors than physical activity (Kind et al., 2019). Therefore, for an aerobic endurance measurement, the PER was used to calculate the predicted maximal oxygen consumption (VO_2max_) with the formula developed by Wyss (Wyss et al., 2019): Predicted VO_2max_ [mL/kg min] = 0.02175 ^∗^ PER [s] + 33.29. The SLJ was used as a muscle power measurement because it correlates most with the overall body explosive power (Fraser et al., 2017). In addition, to the physical test, an assessment of the physical activity behavior (leisure and job-related) and an index for sport-related intentions was made using a self-report questionnaire. As described previously by Wyss (Wyss et al., 2019), this included the International Physical Activity Questionnaire in short form (IPAQ short) and additional statements on sport related intentions. The index for sport-related intentions (Motivation Score) has a range of 0–60 points and the recruits were divided into 5 categories of physical activity based on the score achieved in the IPAQ short (Wyss et al., 2019).

### Linkage

Because there was no unique ID number in both our data sets, we individually linked the fitness test data described above to the medical examination data (both exports from the same data base) using identifiers present in both data sets, height, weight, recruitment center, date of data base entry (conscription ±10 days) and the overall fitness test result. Linkage was successful for 90.6% individuals (see Supplementary Figure S1, flowchart). We assume that the main reason for the missing links was a time interval larger than ±10 days between data base entry for the fitness test and the medical results (despite being measured on the same day).

### Imputation

In order to handle missing values for VO_2max_/endurance (missingness 27.8%), Standing Long Jump/power (27.8%), creatinine (30.8%), hemoglobin (31.2%), CRP (30.8%), motivation score (28.1%) and physical activity behavior (28.1%), multiple imputation with chained equations (MICE) was applied to impute the incomplete data (van Buuren, 2012). The imputation model includes the incomplete variables and the complete variables: residential altitude, BMI, year of recruitment, day of recruitment, recruitment center, SEP group, urbanicity, age group (Moons et al., 2006). Since the maximum fraction of missing information was approximately 31.2%, we created 31 complete datasets with 25 iterations (Bodner, 2008; White et al., 2011). For each dataset, the analyses were performed separately and merged afterward using Rubin’s rule (Rubin, 2004). We compared the results with a complete-case analysis (Sterne et al., 2009). The results of the multiple imputation and complete-cases analysis differ slightly but lead to the same conclusion. For that reason, only the results of the multiple imputation are shown in the main manuscript. The results of the complete-case analysis, however, can be found in the Supplementary Material.

### Statistical Analysis

General additive model (GAM) framework was used to estimate the (potentially) non-linear association between residential altitude and VO_2max_/Endurance and Standing Long Jump/Power (Wood, 2017). GAM models are an extension of generalized linear models (GLM) obtained by allowing not only for linear associations but also for general smooth terms. Therefore non-linear relationships between response and exploratory variables can be fitted (moreover, the smooth terms reduce to essentially linear relationship if the data do not call for non-linearity). In our models we have a combination of linear and smooth terms. We used smooth terms in the following variables: residential altitude, BMI, creatinine, hemoglobin, day of recruitment (to adjust for seasonality), and linear terms in these variables: motivation score (continuous), physical activity behavior categories, CRP categories, year of recruitment (categorical), recruitment center, SEP groups, urbanicity categories, age groups. We tested all linear terms for multicollinearity by calculating the variance inflation factor and the smoothed terms by measuring the concurvity. All variance inflation factors were smaller than 1.5 and the concurvity indices were smaller than 0.15. Thereby all variables are considered non-collinear.

To examine the importance of each exploratory variable on the response, we removed each exploratory variable from the model separately and calculated the AIC (Akaike’s information criterion). Next, the difference of each AIC model under omission of one exploratory variable and the full model were calculated and ordered. The larger the AIC, the more important the exploratory variable in the model is.

All statistical analyses were performed using R Version 3.6.0. The R package “mice” (van Buuren and Groothuis-Oudshoorn, 2011) was used to impute the missing data and the package “mgcv” (Wood, 2017) for the generalized additive models. We used ggplot2 (Wickham, 2016) to produce all figures.

## Results

A total of *N* = 108,677 young men were included in the study (see Supplementary Figure S1, flowchart). Mean BMI was 23.3 kg/m^2^, the prevalence of excess weight (BMI >= 25.0 kg/m^2^) was 23.6%, and all other descriptive statistics are presented in the SDC (see Supplementary Table S1, descriptives). The majority of the young men (60.5%, *N* = 66,012) lived between 300 and 599 masl (Table 1). Mean altitude was 616.2 masl, and only 3.3% (*N* = 3,525) young men lived at or above 1200 masl (see also Supplementary Figure S3, histograms). Based on the descriptive analysis, we observed a 3.6% increase in average endurance performance from 50.07 ml/kg min (SD = 4.69) among the lowest residential altitude group (300–599 masl) to 51.87 ml/kg min (SD = 4.45) among the highest residential altitude group (>=1800 masl).

**TABLE 1:** Descriptive statistics of endurance (VO_2max_, [ml/kg min]) and muscle power performance (Standing Long Jump, [meters]) per residential altitude levels per 300 masl, based on the full dataset after multiple imputation.

Residential					Standing long	
altitude	N	N[%]	VO_2max_/endurance		jump/power	
				
Groups [masl]			Mean [ml/kg min]	SD	Mean [meters]	SD
>=1800	271	0.26	51.87	4.45	2.38	0.22
1500–1799	1038	0.97	50.80	4.68	2.32	0.25
1200–1499	2216	2.07	51.01	4.66	2.32	0.25
900–1199	7019	6.50	50.62	4.62	2.30	0.24
600–899	32121	29.70	50.52	4.58	2.31	0.23
300–599	66012	60.50	50.07	4.69	2.31	0.23

*(masl, meters above sea level; N, absolute frequency; N [%], percentage of absolute frequency, SD, standard deviation).*

**FIGURE 1:**
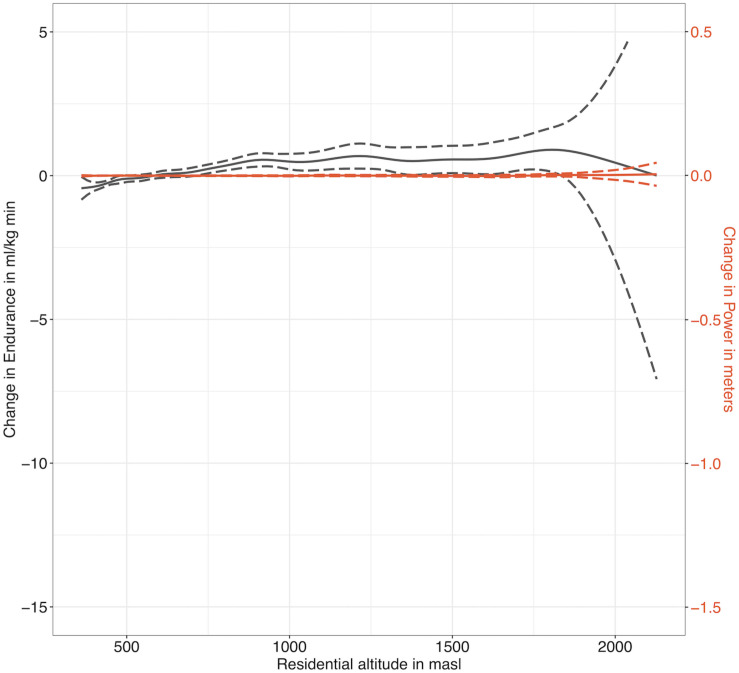
Smoothed association between residential altitude and endurance (VO_2max_ in black) as well as muscle power performance (Standing Long Jump in red) from adjusted GAM models, based on the full dataset after multiple imputation (dashed lines = 95% confidence intervals, masl = meters above sea level, the scales of the primary and secondary axes are chosen according to Figure 2 in order to ensure comparability).

When modeling exclusively the association between residential altitude and either, endurance or muscle power performance, we observe a significant positive association with both (see Supplementary Table S2, GAM model descriptives). However, when adjusting the GAM models for all available co-factors, the significant association persisted for endurance (*p* < 0.001) but not for muscle power performance (*p* = 0.858) (see Supplementary Table S2, GAM model descriptives). The visualizations of the adjusted smoothed terms reveal that there was a significant increase in endurance performance until about 1200 masl, whereas there was no association with muscle power performance (Figure 1). The fully adjusted GAM models for endurance explained 43.9% of deviance in endurance performance, while deviance explained in muscle power performance was 29.1%.

**FIGURE 2:**
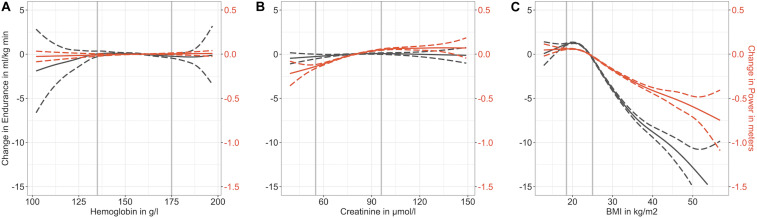
GAM models with smoothed term associations between Hb [g/l] **(A)**, creatinine [μmol/l] **(B)**, and BMI [kg/m^2^] **(C)** on the one hand, and endurance (VO_2max_ in black) and muscle power performance (Standing Long Jump in red) on the other hand, based on the full dataset after multiple imputation (dashed lines = 95% confidence intervals, gray vertical lines = medical norm thresholds according to the Viollier laboratory lower respectively, upper thresholds: hemoglobin 135 resp. 175 g/l, creatinine 55 resp. 96 μmol/l, BMI 18.5 resp. 25.0 kg/m^2^).

When rating the explanatory variables from the fully adjusted GAM models according to their contribution to AIC (Table 2), we observe that BMI had the greatest association with both, endurance and muscle power performance. The motivation score and physical activity behavior were also important contributors for both endurance and muscle power performance. For endurance performance, residential altitude was an important covariate (even when adjusting for Hb in the model), however, it was not important for muscle power performance. We also observed that creatinine contributed strongly for muscle power performance but not for endurance performance. Inflammation and Hb both were stronger contributors in explaining endurance than muscle power performance.

**TABLE 2:** Rating of explanatory factors from the adjusted endurance (VO_2max_) and muscle power performance (Standing Long Jump) GAM models sorted according to contribution to AIC (Akaike’s information criterion), based on the full dataset after multiple imputation.

Rank	VO_2max_/endurance		Rank	Standing long jump/power	
			
Importance	Term	Delta AIC	Importance	Term	Delta AIC
1.	s(BMI)	27713.6	1.	s(BMI)	18355.4
2.	Motivation Score	11834.1	2.	Motivation Score	5958.1
3.	Physical Activity Behavior	6320.1	3.	s(Creatinine)	4765.4
4.	s(Residential Altitude)	616.8	4.	Physical Activity Behavior	995.3
5.	CRP	285.2	5.	Recruitment Center	829.7
6.	Age	267.1	6.	Year	209.4
7.	SEP	221.0	7.	SEP	116.1
8.	Recruitment Center	145.5	8.	Urbanicity	100.7
9.	Days	111.5	9.	Age	66.6
10.	Year	104.3	10.	CRP	48.4
11.	s(Hemoglobin)	101.9	11.	Days	46.3
12.	Urbanicity	92.2	12.	s(Residential Altitude)	13.4
13.	s(Creatinine)	47.4	13.	s(Hemoglobin)	10.1

*(delta AIC = difference between AIC with the respective deleted term deleted and the AIC of the full model, s(x) = smooth, potentially non-linear term in the variable x).*

The visualized smoothed term associations between Hb, creatinine and BMI on the one hand and endurance and muscle power performance on the other hand reveal notable non-linearities. Low Hb tends to show a negative association with endurance performance, but revealed no association with muscle power performance. Conversely, low creatinine is associated with muscle power performance, but there was no association with endurance performance. Whereas a higher BMI showed a negative association with both endurance and muscle power performance, underweight revealed a slight negative association with endurance but not with muscle power performance. Of note, performance in endurance drops at a stronger rate with increasing BMI than the performance in muscle power. The complete case data analysis produced similar results (see Supplementary Tables S3–S6 and Supplementary Figures S4–S6).

## Discussion

This Swiss study assesses the association between residential altitude and endurance and muscle power performance among the general population of young Swiss men. We show that an increase in residential altitude was associated with increased endurance but not with muscle power performance. We also find a strong association between BMI, motivation score, and general physical activity behavior and performance. Blood parameters such as Hb, creatinine and CRP increase the explanatory power of the models.

Previous studies on the physical fitness performance of Swiss conscripts between 2006 and 2015 based on the same sports data have shown that among the investigated population, 73.8% fulfilled basic physical activity recommendations [which corresponds to the Swiss general population (Federal Office of Public Health, 2019)] and that aerobic endurance and muscle power did not show temporal changes over time (Wyss et al., 2019). While BMI, motivation, physical activity behavior, urbanicity, and occupational background have already been identified as important determinants of physical performance in earlier studies (Wyss et al., 2019), here we show that residential altitude and blood parameters increase the explanatory power of the models. We partially confirm our hypothesis and show that residential altitude was associated with determinants of cardiorespiratory fitness (VO_2max_). However, we were not able to control our analysis for known covariates, such as smoking behavior and baseline heart rate, which influence aerobic endurance (Kind et al., 2019) as this information was not included in the personal data. A strong association of serum creatinine-levels with strength performance has also been found in the general United States population (Fragala et al., 2017). Similarly, the association between Hb and endurance performance followed the expected direction (Kanstrup and Ekblom, 1984).

We found a significant association of altitude on endurance performance even after adjustment for Hb, creatinine, BMI and sociodemographic factors. We propose that unobserved factors might explain the remaining association between altitude and endurance performance. Such speculative factors include: (i) higher UV exposure and consequently lesser Vitamin D deficiency at higher residential altitude may be accompanied by improved muscle health (Faeh et al., 2016); (ii) less air pollution or lower carbon monoxide levels at higher altitudes may further lead to improved lung function and therefore increased aerobic endurance (Ackermann-Liebrich et al., 1997; Faeh et al., 2016); (iii) lifestyle factors, for which we cannot control for in our data, may also play a role. For instance, smoking habits (Kind et al., 2019), and/or regional nutritional patterns may vary depending on someone’s residential altitude. Interestingly, we observed the positive association between residential altitude and endurance performance only up to 1200 masl. Perhaps, this phenomenon depicts an “upper limit” or “saturation-type” relation.

It is also conceivable that genetic differences at least partially account for the remaining association between altitude and endurance performance (Wittwer et al., 2004). Based on the observation of our earlier work that Hb values rose in these conscripts every 300 m of increase in altitude, we postulated that the cellular oxygen-sensing system is constitutively activated already at moderate altitude (Staub et al., 2020). This in turn might stabilize the α-subunits of the hypoxia-inducible factors (HIFs), especially of HIF-2 that is responsible for the adaptation to chronic exposure to hypoxia (van Patot and Gassmann, 2011). Indeed, HIF-2 orchestrates expression of hundreds of hypoxia-induced genes. Even more so, as it has been shown that several ethnicities developed different adaptations when exposed to varying altitudes (Gassmann et al., 2019).

It is also possible that biological variability and heterogeneity in subpopulations in higher residential areas is somewhat smaller (Staub et al., 2018). This has already been shown for Hb (fewer low values at higher altitudes) (Sharma et al., 2019; Staub et al., 2020). However, in the case of endurance and muscle power performance, the standard deviations do not show major changes with increasing residential altitude (Table 1). However, such potential evolutionary aspects should be considered in future studies. The finding that the deviance explained for endurance is higher than for muscle power may also be explained by the fact that technique (and thus training) is more important for SLJ than for running.

The second part of our hypothesis (muscle power performance increases as well with rising residential altitude) was rejected, at least after adjusting for all other co-factors. There are not many studies with which we could compare our data, but the inconsistent results could also be due to different test batteries for muscle power or different altitude levels (Feriche et al., 2017). More generally speaking, a wider test battery on the medical side (lung function, smoking behavior, additional blood parameters, such as testosterone, vitamin D, and further hormonal levels, etc.) would allow more comprehensive models to be fitted to give more or less weight to some of the speculations made above.

### Strengths and Limitations of the Study

One of the strengths of this study is its large population sample, enabled by the high coverage of the Swiss conscription database (>90% of a given male birth cohort is included). The data also include those conscripts who were later declared unfit for military service. We only included young men and also only those who have Swiss citizenship. This is for example also true for Hb values varying altitudes that are ethnicity-specific (Gassmann et al., 2019). It is highly desirable to identify and analyze similar cohorts worldwide in future studies. Motivation is very important for sport performance among the general population. We can only approximate this aspect by adjusting for the motivation score in our data, but some motivational effect may still remain. In future studies, it would be interesting to investigate a possible interplay between BMI (as a physical predisposition) and motivation score (as a psychological factor) – we can only speculate that some compensation can occur (and worse BMI might be counterbalanced by better motivation to some extent).

In our data, the conscripts’ officially listed place of residence was the only reference point available. Thus, it is unknown how long the conscripts were present at their place of residence prior to conscription (unknown residential history). Also, we cannot control for potential longer stays at higher or lower altitude immediately before conscription, as such changes of altitudes can alter endurance performance (Saunders et al., 2009). This can lead to some distortion. In particular, students may be listed at their parents’ place of residence but spend their daily lives elsewhere, such as near a university in a larger Swiss city. We have taken this possible inaccuracy into account by controlling our models for the professional status of young men, with which we identify pupils/students as a separate group. Another limitation is that the data did not provide information on the type of physical activity and sports performed by the conscripts. Although we picture work and everyday life related physical activity to some degree via our variable physical activity behavior (which is based on the IPAQ short), we cannot rule out that especially steadily job and everyday life low-dose physical activity levels may change slightly depending on residential altitudes, which could also influence endurance performance. We did not examine the role of specific job profiles (e.g., farm workers) on different residential altitude. It has been shown, that in subsistence populations like the Tsimane of Bolivia, high everyday life physical activity levels lead to higher VO_2max_ levels and few indicators of cardiovascular disease (Pisor et al., 2013). Such associations and potential parallels with subpopulations living at higher altitudes in Switzerland should be looked at more closely in future studies. Moreover, it is obvious that with increasing residential altitude, the number of observations becomes smaller. Although Switzerland has some high-altitude areas, the country’s residential altitude is limited predominantly to moderate and low altitude. Also note that the BMI is a somewhat inaccurate measure of excess weight and body shape, as it is based solely on height and weight (which may also be muscle-related). From 2019 onward, future studies on these conscription data will also be able to draw on waist circumference measurements, which have currently been introduced as a standard measurement in the recruitment process.

## Conclusion

By showing that a modest increase in residential altitude is associated with better endurance performance (but not with muscle power performance), we enhance knowledge of physical activity capacity from a public health perspective. About a quarter of young men in Switzerland do not comply with the basic physical activity recommendations. This may have a major and potentially long-standing impact on the health of these individuals later in life and might increase economic burden on the health care system. Thus, monitoring the health and fitness status of young people and its determinants is important for a precisely targeted disease prevention and the public health system. The earlier risk groups can be identified and refined prevention programs launched, the more these long-term negative effects can be reduced. Here we show that there is added value in combining different data sets (here: medical, sports performance, and residential altitude data) to pursue this long-term goal. However, future studies should specifically investigate other potential explanations for the remaining association between residential altitude and endurance performance that, in our study remains important even after adjusting for other currently available variables.

## Data Availability Statement

The datasets generated for this study will not be made publicly available. The data is owned by the Swiss Armed Forces. Based on the data contract, the authors are not allowed to share the individual data. However, upon submission of a study protocol and signing a data contract the Swiss Armed Forces can make the fully anonymized individual data available for other interested researchers.

## Ethics Statement

The Swiss Armed Forces (Armed Forces Staff) provided the data and gave permission to analyze it. The records were fully anonymized by the Swiss Armed Forces. All exact residential addresses, social security numbers, exact birthdates and names were removed before the data was provided. This study uses anthropometric and laboratory governmental data, which is defined as nonclinical, governmental data. The Swiss Armed Forces has the authorization to provide anonymous data for academic research according to Swiss federal law (Bundesgesetz ber die militärischen Informationssysteme MIG, BG 510.91, Art. 2, 9, 24–29). Hence there is no need for further ethical approval (Swiss data privacy act, SR 235.1; 19.6.1992 and Federal Act on Research involving Human Beings HRA, 810.30; 1.1.2014) (Panczak et al., 2014; Bruggisser et al., 2016). The detailed informed consent form allowing the voluntary laboratory test signed by all conscripts can be requested from the Swiss Armed Forces.

## Author Contributions

KS, FR, and TW obtained data from the Swiss Armed Forces. KS and FR contributed to the supervision. NG, KM, KS, and MB conceived the data presentation. NG, KM, and KS designed the data presentation. MZ and RP prepared the SNC altitude data. KM analyzed the data. NG and KS wrote first draft. All authors wrote the manuscript. KS and FR obtained funding.

## Conflict of Interest

The authors declare that the research was conducted in the absence of any commercial or financial relationships that could be construed as a potential conflict of interest.

## Footnotes

1 www.swisstopo.ch

2 www.swissnationalcohort.ch
