# Supplementary material for: Residential Altitude Associates With Endurance but Not Muscle Power in Young Swiss Men
Source: Front Physiol. 2020 Jul 23;11:860. doi: 10.3389/fphys.2020.00860 (PMC7390881; doi:10.3389/fphys.2020.00860)

Supplemental Digital Content (SDC)

SDC Table 1: Overall descriptive statistics, based on the full dataset after multiple imputation. (N = absolute frequency; SD = standard deviation).

	**Overall (N = 108677)**
**VO2max/Endurance [ml/kg min]**	
Mean (SD)	50.3 (4.7)
Range	21.7 - 70.3
**Standing Long Jump/Power [meters]**	
Mean (SD)	2.3 (0.2)
Range	0.2 - 3.2
**Creatinine [µmol/l]**	
Mean (SD)	82.0 (12.0)
Range	39.0 - 149.0
**Hemoglobin [g/l]**	
Mean (SD)	157.1 (8.7)
Range	102.0 - 197.0
**Residential Altitude [masl]**	
Mean (SD)	616.2 (228.9)
Range	360.7 - 2127.5
**BMI [kg/m2]**	
Mean (SD)	23.3 (3.8)
Range	12.8 - 56.8
**Motivation Score [points]**	
Mean (SD)	43.4 (11.2)
Range	0.0 - 60.0
**Year**	
2007	18035 (16.6%)
2008	17540 (16.1%)
2009	18124 (16.7%)
2010	18667 (17.2%)
2011	18305 (16.8%)
2012	18006 (16.6%)
**Recruitment Center**	
Lausanne	45204 (41.6%)
Sumiswald	34359 (31.6%)
Mels	29114 (26.8%)
**SEP Groups**	
High	35652 (32.8%)
Imprecise	8950 (8.2%)
Low	28915 (26.6%)
Medium	27661 (25.5%)
Pupil	7499 (6.9%)
**Physical Activity Behaviour Categories**	
inactive individuals	13021 (12.0%)
partially active individuals	18244 (16.8%)
irregularly active individuals	22761 (20.9%)
regularly active individuals	6924 (6.4%)
regularly vigorously active individuals	47727 (43.9%)
**Urbanicity**	
non-urban	66226 (60.9%)
urban	42451 (39.1%)
**Age Groups [years]**	
[18-19)	26174 (24.1%)
[19-20)	50797 (46.7%)
[20-21)	23424 (21.6%)
[21-22)	8282 (7.6%)
**CRP [mg/l]**	
< 5.0	101686 (93.6%)
>= 5.0	6991 (6.4%)

SDC Table 2: Descriptive table of GAM model for unadjusted and adjusted effects of residential altitude on endurance and muscle power performance, based on the full dataset after multiple imputation. (edf = effective degrees of freedom; SE = standard error, s(x) = smooth, potentially nonlinear term in the variable x).

	edf	F	p.value
**Unadjusted Residential Altitude VO2max/Endurance**			
s(Residential Altitude)	14.60	33.4	<0.001
**Unadjusted Residential Altitude Standing Long Jump/Power**			
s(Residential Altitude)	18.18	7.3	<0.001
**Adjusted VO2max/Endurance smooth**			
s(Residential Altitude)	10.78	38.8	<0.001
s(Hemoglobin)	5.74	5.9	<0.001
s(Creatinine)	2.88	6.3	<0.001
s(BMI)	7.10	3129.5	<0.001
s(Days)	7.14	11.7	<0.001
**Adjusted Standing Long Jump/Power smooth**			
s(Residential Altitude)	18.57	0.7	0.858
s(Hemoglobin)	2.16	3.7	0.014
s(Creatinine)	5.57	432.9	<0.001
s(BMI)	6.23	1976.0	<0.001
s(Days)	8.62	3.5	<0.001

Adjusted VO2max/Endurance linear	Estimate	SE	t.value	p.value
**Intercept**	43.46	0.1	641.4	<0.001
**Motivation Score [points]**	0.13	0.0	96.1	<0.001
**Recruitment Center**				
Lausanne*	-	-	-	-
Sumiswald	0.38	0.0	10.7	<0.001
Mels	0.23	0.0	6.8	<0.001
**SEP Groups**				
Imprecise	-0.62	0.0	-13.1	<0.001
Low	-0.19	0.0	-5.8	<0.001
Medium	-0.20	0.0	-6.0	<0.001
High*	-	-	-	-
Pupil	-0.22	0.1	-4.3	<0.001
**Physical Activity Behavior Categories**				
inactive individuals*	-	-	-	-
partially active individuals	0.51	0.0	10.9	<0.001
irregularly active individuals	1.27	0.0	26.5	<0.001
regularly active individuals	1.51	0.1	23.5	<0.001
regularly vigorously active individuals	2.67	0.0	56.6	<0.001
**Year**				
2007*	-	-	-	-
2008	<0.01	0.0	0.0	0.987
2009	-0.15	0.0	-3.4	0.001
2010	-0.21	0.0	-5.0	<0.001
2011	-0.31	0.0	-7.8	<0.001
2012	-0.21	0.0	-5.0	<0.001
**Urbanicity**				
non-urban*	-	-	-	-
urban	-0.23	0.0	-8.7	<0.001
**Age [years]**				
[18-19)*	-	-	-	-
[19-20)	-0.18	0.0	-6.0	<0.001
[20-21)	-0.39	0.0	-10.8	<0.001
[21-22)	-0.67	0.1	-12.1	<0.001
**CRP [mg/l]**				
<5.0*	-	-	-	-
>=5.0	-0.71	0.1	-12.7	<0.001

* Reference

Adjusted Standing Long Jump/Power linear	Estimate	SE	t.value	p.value
**Intercept**	2.05	0.0	524.3	<0.001
**Motivation Score [points]**	0.01	0.0	70.8	<0.001
**Recruitment Center**				
Lausanne*	-	-	-	-
Sumiswald	0.01	0.0	7.9	<0.001
Mels	0.05	0.0	27.0	<0.001
**SEP Groups**				
Imprecise	-0.02	0.0	-8.5	<0.001
Low	-0.01	0.0	-4.9	<0.001
Medium	<0.01	0.0	1.6	0.121
High*	-	-	-	-
Pupil	-0.01	0.0	-4.2	<0.001
**Physical Activity Behavior Categories**				
inactive individuals*	-	-	-	-
partially active individuals	0.01	0.0	4.3	<0.001
irregularly active individuals	0.03	0.0	11.7	<0.001
regularly active individuals	0.04	0.0	12.6	<0.001
regularly vigorously active individuals	0.06	0.0	22.8	<0.001
**Year**				
2007*	-	-	-	-
2008	<0.01	0.0	-0.5	0.631
2009	-0.02	0.0	-7.6	<0.001
2010	-0.02	0.0	-10.6	<0.001
2011	-0.02	0.0	-10.3	<0.001
2012	-0.02	0.0	-8.7	<0.001
**Urbanicity**				
non-urban*	-	-	-	-
urban	-0.01	0.0	-8.4	<0.001
**Age [years]**				
[18-19)*	-	-	-	-
[19-20)	0.01	0.0	4.5	<0.001
[20-21)	0.02	0.0	7.3	<0.001
[21-22)	0.01	0.0	3.5	<0.001
**CRP [mg/l]**				
<5.0*	-	-	-	-
>=5.0	-0.02	0.0	-4.5	<0.001

SDC Table 3: Descriptive statistics of endurance (VO_{2max}, [ml/kg min]) and muscle power performance (Standing Long Jump, [meters]) per residential altitude levels per 300 masl, based on the complete case dataset. (masl = meters above sea level; N = absolute frequency; N [%] = percentage of absolute frequency, SD = standard deviation).

Residential Altitude Groups [masl]	N	N [%]	VO2max/Endurance Mean [ml/kg min]	SD	Standing Long Jump/Power Mean [meters]	SD
>=1800	153	0.28	51.99	4.21	2.40	0.22
1500-1799	529	0.95	50.99	4.56	2.34	0.26
1200-1499	1162	2.09	51.34	4.43	2.34	0.24
900-1199	3710	6.64	51.02	4.45	2.31	0.23
600-899	17266	30.84	50.92	4.37	2.32	0.22
300-599	33437	59.20	50.46	4.52	2.32	0.22

SDC Table 4: Overall descriptive statistics, based on the complete case dataset. (N = absolute frequency; SD = standard deviation).

	Overall (N=56257)
**VO2max/Endurance [ml/kg min]**	
Mean (SD)	50.7 (4.5)
Range	31.2 - 70.3
**Standing Long Jump/Power [meters]**	
Mean (SD)	2.3 (0.2)
Range	0.2 - 3.2
**Creatinine [µmol/l]**	
Mean (SD)	81.9 (11.9)
Range	42.0 - 149.0
**Hemoglobin [g/l]**	
Mean (SD)	156.9 (8.7)
Range	102.0 - 197.0
**Residential Altitude [masl]**	
Mean (SD)	620.1 (229.6)
Range	360.7 - 1989.3
**BMI [kg/m2]**	
Mean (SD)	23.2 (3.4)
Range	15.0 - 48.9
**Motivation Score [points]**	
Mean (SD)	44.3 (10.8)
Range	0.0 - 60.0
**Year**	
2007	8343 (14.8%)
2008	7860 (14.0%)
2009	8295 (14.7%)
2010	10794 (19.2%)
2011	10540 (18.7%)
2012	10425 (18.5%)
**Recruitment Center**	
Lausanne	22741 (40.4%)
Sumiswald	16435 (29.2%)
Mels	17081 (30.4%)
**SEP Groups**	
High	18189 (32.3%)
Imprecise	4095 (7.3%)
Low	15034 (26.7%)
Medium	15300 (27.2%)
Pupil	3639 (6.5%)
**Physical Activity Behaviour Categories**	
inactive individuals	5961 (10.6%)
partially active individuals	8975 (16.0%)
irregularly active individuals	11912 (21.2%)
regularly active individuals	3590 (6.4%)
regularly vigorously active individuals	25819 (45.9%)
**Urbanicity**	
non-urban	34934 (62.1%)
urban	21323 (37.9%)
**Age Groups [years]**	
[18-19)	14273 (25.4%)
[19-20)	26717 (47.5%)
[20-21)	11496 (20.4%)
[21-22)	3771 (6.7%)
**CRP [mg/l]**	
< 5.0	52850 (93.9%)
>= 5.0	3407 (6.1%)

SDC Table 5: Descriptive table of GAM model for unadjusted and adjusted effects of residential altitude on endurance and muscle power performance, based on the complete case dataset. (edf = effective degrees of freedom; SE = standard error, s(x) = smooth, potentially nonlinear term in the variable x).

	edf	F	p.value
**Unadjusted Residential Altitude VO2max/Endurance**			
s(Residential Altitude)	14.33	23.3	<0.001
**Unadjusted Residential Altitude Standing Long Jump/Power**			
s(Residential Altitude)	14.97	5.0	<0.001
**Adjusted VO2max/Endurance smooth**			
s(Residential Altitude)	14.10	23.5	<0.001
s(Hemoglobin)	6.24	13.9	<0.001
s(Creatinine)	2.42	5.1	0.002
s(BMI)	7.91	1892.6	<0.001
s(Days)	7.93	11.2	<0.001
**Adjusted Standing Long Jump/Power smooth**			
s(Residential Altitude)	7.92	1.6	0.109
s(Hemoglobin)	2.13	2.7	0.049
s(Creatinine)	4.40	518.9	<0.001
s(BMI)	6.25	1285.0	<0.001
s(Days)	8.69	7.1	<0.001

Adjusted VO2max/Endurance linear	Estimate	SE	t.value	p.value
**Intercept**	44.10	0.1	523.2	<0.001
**Motivation Score [points]**	0.13	0.0	77.8	<0.001
**Recruitment Center**				
Lausanne*	-	-	-	-
Sumiswald	0.24	0.0	6.0	<0.001
Mels	0.12	0.0	3.1	0.002
**SEP Groups**				
Imprecise	-0.47	0.1	-8.0	<0.001
Low	-0.20	0.0	-5.1	<0.001
Medium	-0.21	0.0	-5.3	<0.001
High*	-	-	-	-
Pupil	-0.14	0.1	-2.1	0.032
**Physical Activity Behavior Categories**				
inactive individuals*	-	-	-	-
partially active individuals	0.44	0.1	7.6	<0.001
irregularly active individuals	1.19	0.1	20.5	<0.001
regularly active individuals	1.41	0.1	19.0	<0.001
regularly vigorously active individuals	2.53	0.1	44.3	<0.001
**Year**				
2007*	-	-	-	-
2008	-0.01	0.1	-0.2	0.847
2009	-0.18	0.1	-3.3	0.001
2010	-0.16	0.1	-3.3	0.001
2011	-0.26	0.0	-5.3	<0.001
2012	-0.19	0.1	-3.9	<0.001
**Urbanicity**				
non-urban*	-	-	-	-
urban	-0.19	0.0	-6.2	<0.001
**Age [years]**				
[18-19)*	-	-	-	-
[19-20)	-0.20	0.0	-5.7	<0.001
[20-21)	-0.44	0.0	-10.1	<0.001
[21-22)	-0.79	0.1	-12.4	<0.001
**CRP [mg/l]**				
<5.0*	-	-	-	-
>=5.0	-0.68	0.1	-11.3	<0.001

* Reference

Adjusted Standing Long Jump/Power linear	Estimate	SE	t.value	p.value
**Intercept**	2.08	0.0	433.2	<0.001
**Motivation Score [points]**	<0.01	0.0	51.9	<0.001
**Recruitment Center**				
Lausanne*	-	-	-	-
Sumiswald	0.01	0.0	4.6	<0.001
Mels	0.04	0.0	19.4	<0.001
**SEP Groups**				
Imprecise	-0.02	0.0	-5.8	<0.001
Low	-0.01	0.0	-3.7	<0.001
Medium	<0.01	0.0	1.2	0.240
High*	-	-	-	-
Pupil	-0.01	0.0	-1.9	0.057
**Physical Activity Behavior Categories**				
inactive individuals*	-	-	-	-
partially active individuals	0.01	0.0	2.9	0.004
irregularly active individuals	0.03	0.0	8.4	<0.001
regularly active individuals	0.04	0.0	9.7	<0.001
regularly vigorously active individuals	0.05	0.0	16.8	<0.001
**Year**				
2007*	-	-	-	-
2008	<0.01	0.0	0.0	0.990
2009	-0.03	0.0	-9.7	<0.001
2010	-0.03	0.0	-11.0	<0.001
2011	-0.03	0.0	-9.1	<0.001
2012	-0.02	0.0	-7.8	<0.001
**Urbanicity**				
non-urban*	-	-	-	-
urban	-0.01	0.0	-5.9	<0.001
**Age [years]**				
[18-19)*	-	-	-	-
[19-20)	0.01	0.0	3.6	<0.001
[20-21)	0.01	0.0	5.1	<0.001
[21-22)	<0.01	0.0	1.3	0.210
**CRP [mg/l]**				
<5.0*	-	-	-	-
>=5.0	-0.02	0.0	-4.6	<0.001

SDC Table 6: Rating of explanatory factors from the adjusted endurance (VO_{2max}) and muscle power performance (Standing Long Jump) GAM models sorted according to contribution to AIC (Akaike's information criterion), based on the complete case dataset. (delta AIC = difference between AIC with the respective deleted term and the AIC of the full model, s(x) = smooth, potentially nonlinear term in the variable x).

**Rank Importance**	**VO2max/Endurance Term**	**delta AIC**
**1.**	s(BMI)	14331.3
**2.**	Motivation Score	5743.3
**3.**	Physical Activity Behavior	3117.2
**4.**	s(Residential Altitude)	363.3
**5.**	Age	191.5
**6.**	CRP	125.8
**7.**	s(Hemoglobin)	90.5
**8.**	Days	87.3
**9.**	SEP	69.2
**10.**	Urbanicity	34.4
**11.**	Year	33.8
**12.**	Recruitment Center	32.6
**13.**	s(Creatinine)	13.3

**Rank Importance**	**Standing Long Jump/Power Term**	**delta AIC**
**1.**	s(BMI)	8716.1
**2.**	s(Creatinine)	2710.1
**3.**	Motivation Score	2627.9
**4.**	Physical Activity Behavior	440.8
**5.**	Recruitment Center	401.5
**6.**	Year	225.3
**7.**	SEP	50.4
**8.**	Days	47.4
**9.**	Urbanicity	28.4
**10.**	Age	21.7
**11.**	CRP	18.6
**12.**	s(Residential Altitude)	4.7
**13.**	s(Hemoglobin)	4.5

SDC Figure 1: Flowchart for included data. (N = absolute frequency; [%] = percentage of absolute frequency).

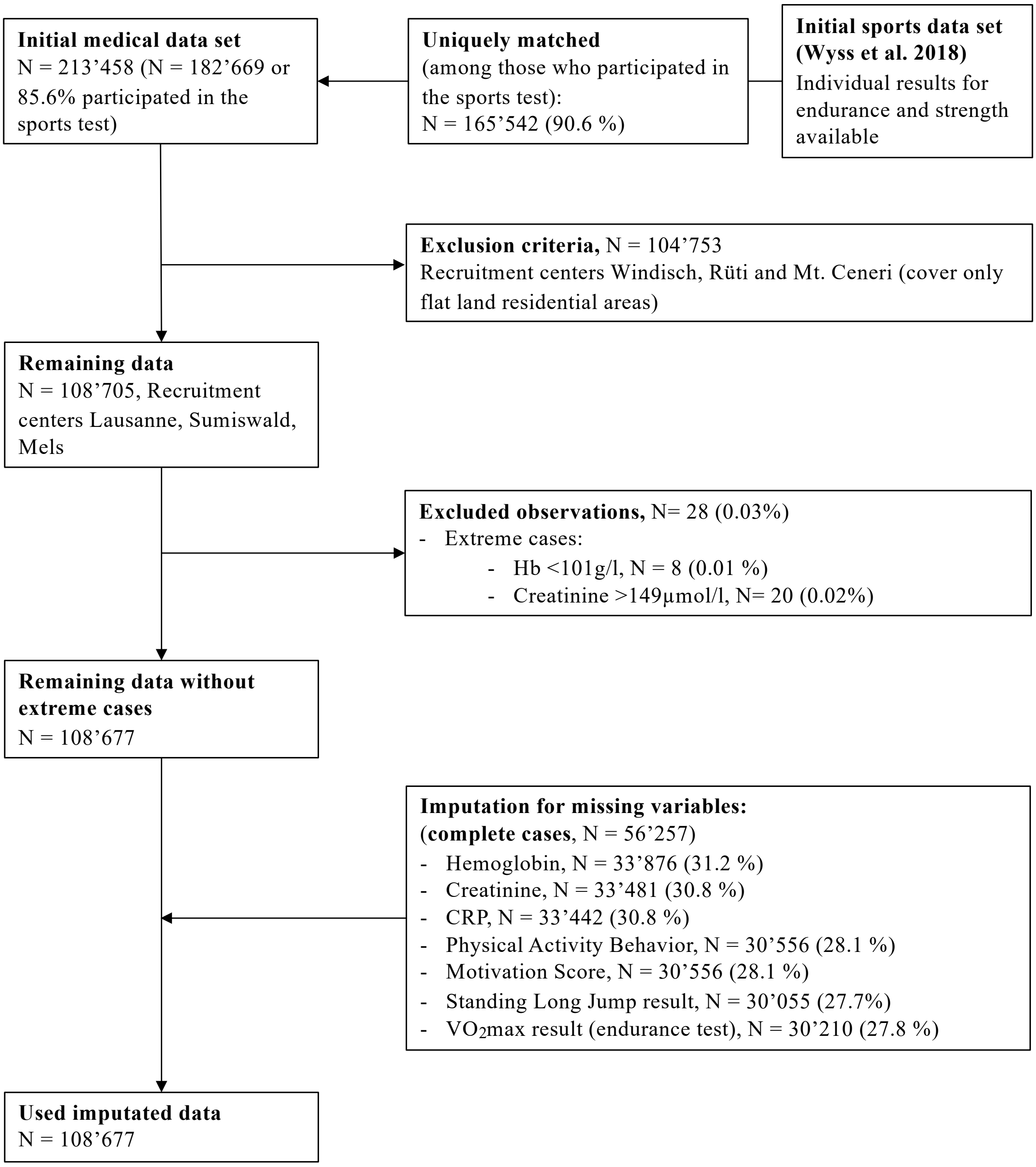

SDC Figure 2: Recruitment center maps of Switzerland. Top: Map of Switzerland showing residential altitude of buildings containing males aged [18-25) per community. Bottom: Allocation of each community to a recruitment center (Lausanne, Sumiswald or Mels). Conscripts of grey shaded communities are not allocated to a specific recruitment center but one of the three recruitment centers that where included in our study. Conscripts of white shaded communities where not allocated to either Lausanne, Sumiswald nor Mels and therefore not included in our study.

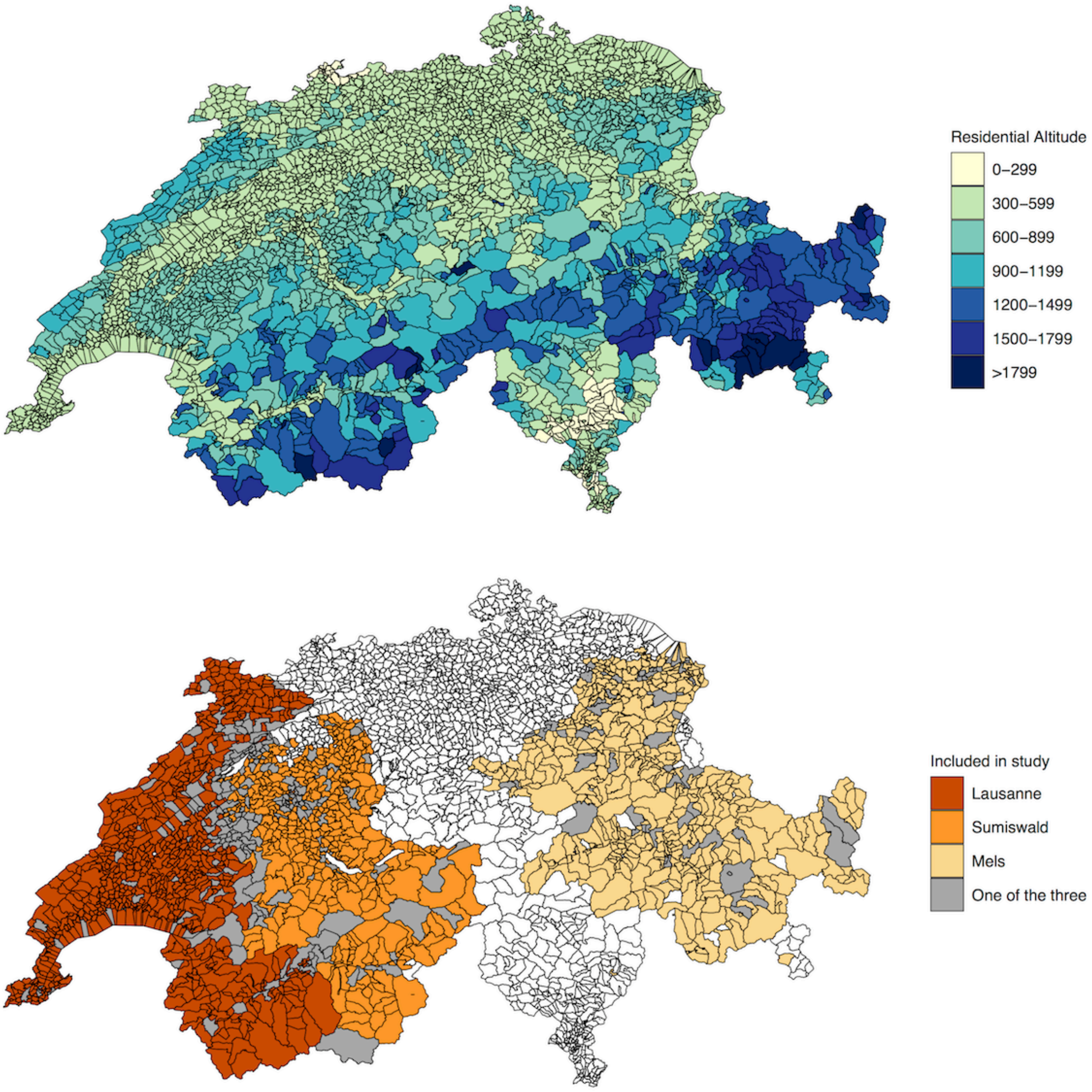

SDC Figure 3: Histograms showing number of conscripts, based on the full dataset after multiple imputation.

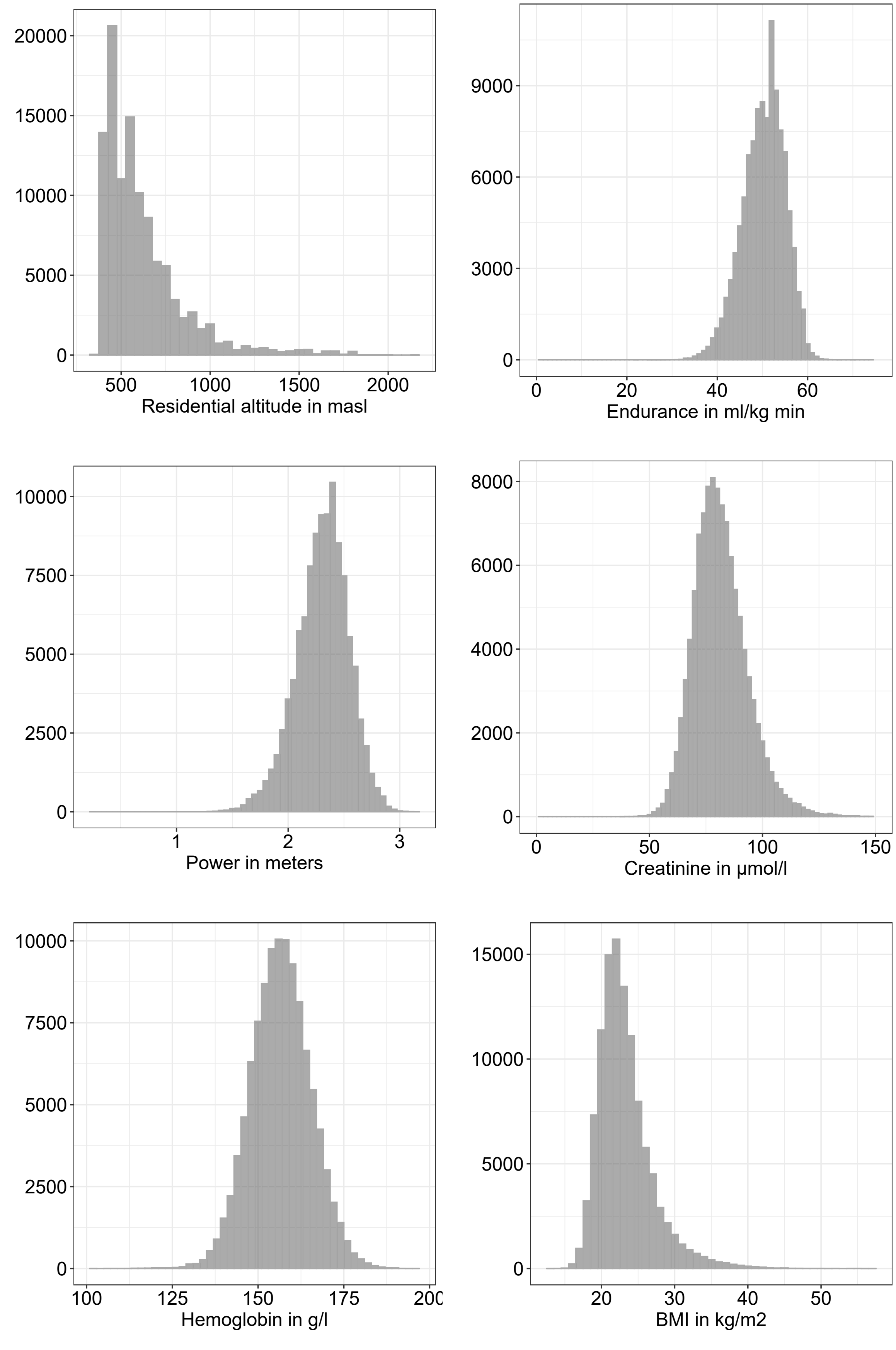

SDC Figure 4: Histograms showing number of conscripts, based on the complete case dataset.

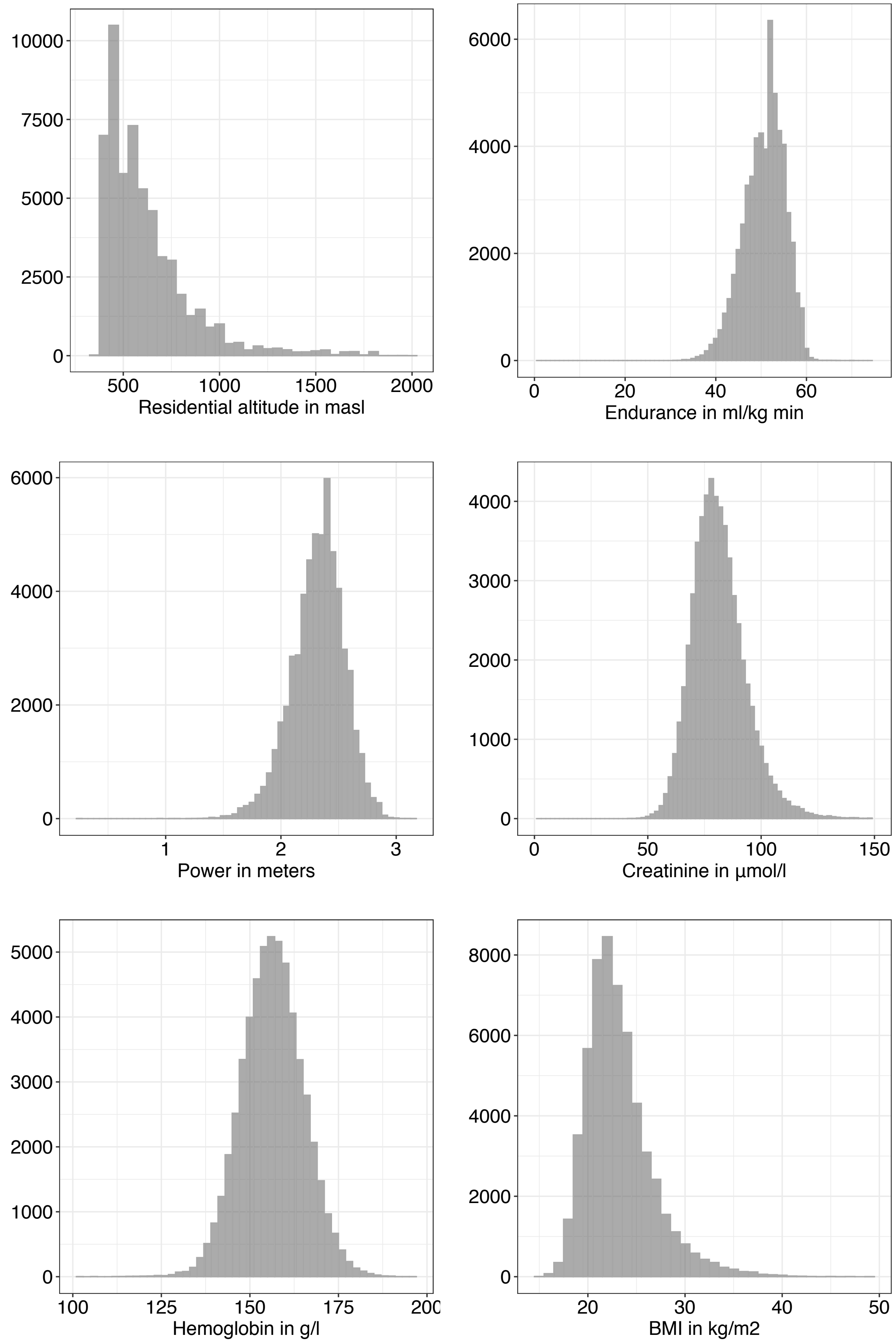

SDC Figure 5: GAM models with adjusted smoothed terms of the change in endurance (VO_{2max} in black) respectively muscle power performance (Standing Long Jump in red) associated with residential altitude, based on the complete case dataset. (dashed lines = 95% confidence intervals, masl = meters above sea level, the scales of the primary and secondary axes are chosen according to Appendix Figure 6 in order to ensure comparability).

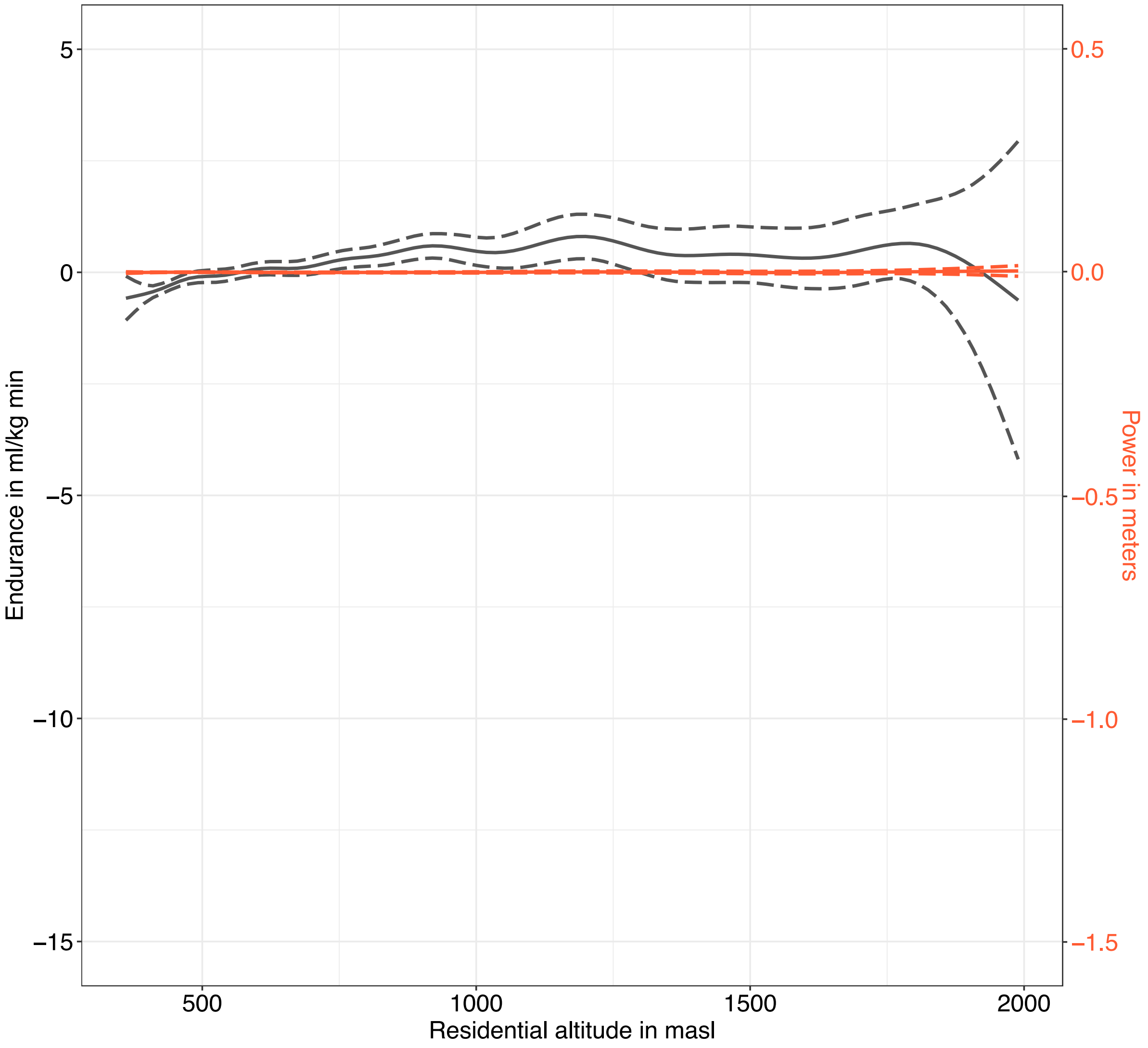

SDC Figure 6: GAM model with smoothed term changes in endurance (VO_{2max} in black) and muscle power performance (Standing Long Jump in red) associated with Hb [g/l] (A), creatinine [µmol/l] (B), and BMI [kg/m^2] (C), based on the complete case dataset. (dashed lines = 95% confidence intervals).

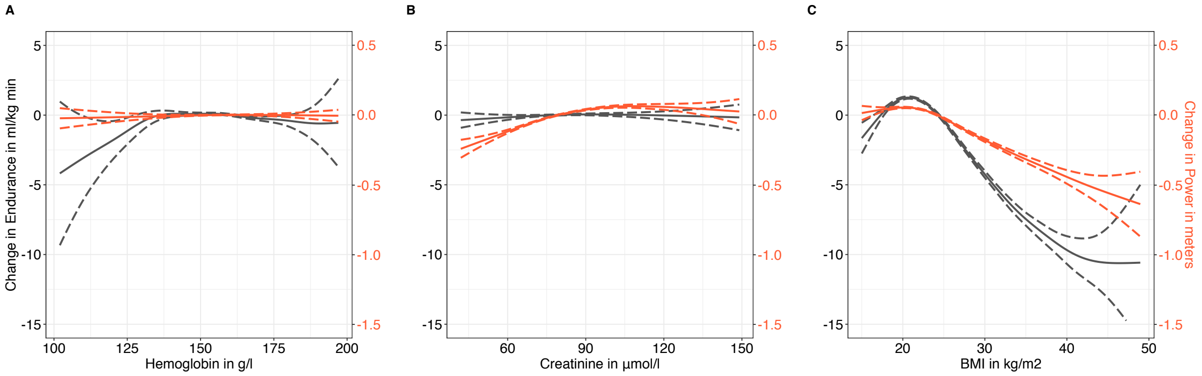

Supplement: Supplementary file 1 [file Presentation_1.pdf]
